# Mortality Risk Among Women With Premenstrual Disorders in Sweden

**DOI:** 10.1001/jamanetworkopen.2024.13394

**Published:** 2024-05-28

**Authors:** Marion Opatowski, Unnur Anna Valdimarsdóttir, Anna Sara Oberg, Elizabeth R. Bertone-Johnson, Donghao Lu

**Affiliations:** 1 Unit of Integrative Epidemiology, Institute of Environmental Medicine, Karolinska Institutet, Stockholm, Sweden; 2 Department of Medical Epidemiology and Biostatistics, Karolinska Institutet, Stockholm, Sweden; 3 Center of Public Health Sciences, Faculty of Medicine, University of Iceland, Reykjavík, Iceland; 4 Department of Biostatistics and Epidemiology, School of Public Health and Health Sciences, University of Massachusetts Amherst, Amherst; 5 Department of Health Promotion and Policy, School of Public Health and Health Sciences, University of Massachusetts Amherst, Amherst

## Abstract

**Importance:**

Premenstrual disorders (PMDs) adversely affect the quality of life of millions of women worldwide, yet research on the long-term consequences of PMDs is limited, and the risk of mortality has not been explored.

**Objective:**

To estimate the associations of PMDs with overall and cause-specific mortality.

**Design, Setting, and Participants:**

This nationwide, population-based, matched cohort study used data from population and health registers in Sweden. Participants included women of reproductive age with a first diagnosis of PMDs between January 1, 2001, and December 31, 2018. Data analysis was performed from September 2022 to April 2023.

**Exposures:**

PMDs were identified through inpatient and outpatient diagnoses and drug dispensing.

**Main Outcomes and Measures:**

Dates of death and underlying causes were ascertained from the National Cause of Death Register. Conditional Cox regression was used to estimate the hazard ratios (HRs) of overall and cause-specific death (eg, death due to natural or nonnatural cause, suicide, or cardiovascular events), adjusting for age, socioeconomic status, and somatic and psychiatric comorbidities; in a separate sibling comparison, models were also adjusted for all factors that sisters share.

**Results:**

A total of 67 748 women with clinically diagnosed PMDs and 338 740 matched unaffected women were included, for a total of 406 488 women. Women with PMDs received a diagnosis at a mean (SD) age of 35.8 (8.2) years. During a mean (SD) follow-up of 6.2 (4.6) years (range, 1-18 years), 367 deaths were observed among women with PMDs (rate, 8.4 deaths per 10 000 person-years; 95% CI, 7.6-9.3 deaths per 10 000 person-years), and 1958 deaths were observed among women without PMDs (rate, 9.1 deaths per 10 000 person-years; 95% CI, 8.7-9.6 deaths per 10 000 person-years). Compared with unaffected women, women with PMDs had increased risk of death due to nonnatural causes (HR, 1.59; 95% CI, 1.25-2.04), particularly suicide (HR, 1.92; 95% CI, 1.43-2.60), but they did not have increased risk of overall mortality (adjusted HR, 0.91; 95% CI, 0.82-1.02). Notably, women who received a diagnosis before the age of 25 years experienced higher all-cause mortality (HR, 2.51; 95% CI, 1.42-4.42) and death from both suicide (HR, 3.84; 95% CI, 1.18-12.45) and natural causes (HR, 2.59; 95% CI, 1.21-5.54).

**Conclusions and Relevance:**

The findings of this matched cohort study suggest that women with PMDs are not at increased risk of early death overall. However, the risk was elevated among young women and for death by suicide. This supports the importance of careful follow-up for young patients and highlights the need to develop suicide prevention strategies for all women with PMDs.

## Key Points

**Question:**

Do women with premenstrual disorders (PMDs) have higher risks of all-cause and cause-specific mortality compared with women without PMDs?

**Findings:**

This nationwide matched cohort study of 406 488 women in Sweden during a mean (SD) follow-up of 6.2 (4.6) years (range, 1-18 years) revealed that overall, women with diagnosed PMDs did not have an increased risk of all-cause premature death. However, there was an increased mortality risk among women with PMDs diagnosed before age 25 years, as well as increased risk of death due to suicide, irrespective of age at diagnosis.

**Meaning:**

These findings suggest that women with PMDs are not at increased risk of all-cause mortality, but active surveillance might be needed among young patients and for suicide prevention for all ages.

## Teaser

This cohort study examines the association of premenstrual disorders with overall and cause-specific mortality among women in Sweden.

## Introduction

Premenstrual disorders (PMDs) are characterized by a range of mental and physical symptoms occurring during the week before menstruation.^1^ These disorders are classified into premenstrual syndrome, which affects 20% to 30% of women of reproductive age, and premenstrual dysphoric disorders, with a prevalence ranging from 2% to 6%.^1,2^ Numerous studies have highlighted the impairments related to PMDs, which can affect women’s daily activities, relationships, and professional performance.^3,4^

Despite the dearth of research on long-term consequences of PMDs, there are some indications of an association between PMDs and mortality. For instance, women with PMDs present with elevated blood pressure levels and a 40% higher risk of developing hypertension.^5,6^ Women with diabetes with PMDs have been found to have a greater risk of uncontrolled blood glucose levels.^7^ In addition, PMDs are highly comorbid with psychiatric disorders,^8,9^ which are associated with elevated risk of both nonnatural and natural-cause mortality.^10,11,12^ Furthermore, we recently showed that, even after accounting for psychiatric comorbidities,^13,14^ women with PMDs were at risk of suicidal behavior and accidents, which are the leading causes of death in young women.^13^ To further our understanding of the risk of death associated with PMDs, this study aimed to evaluate the risks of all-cause and cause-specific mortality among women with clinically diagnosed PMDs and the risks stratified by age groups whenever possible, by leveraging the national health registers in Sweden.

## Methods

### Data Source

This cohort study was approved by the Swedish Ethical Review Authority. Written consent from the participants is not required for register-based studies under Swedish law. This study followed the Strengthening the Reporting of Observational Studies in Epidemiology (STROBE) reporting guidelines for cohort studies.^15^

The study was based on Swedish national health and population registers^16^: (1) the National Patient Register collects all hospital discharge diagnoses in Sweden from 1987 and 80% of hospital-based outpatient visits from 2001, (2) the National Prescribed Drug Register captures redeemed drug prescriptions from all pharmacies since 2005, (3) the National Cause of Death Register comprises death certificates since 1952, (4) the Longitudinal Integration Database for Health Insurance and Labor Market Studies integrates sociodemographic information for all residents aged 16 years and older since 1990, (5) the Total Population Register includes country of birth and migrations, and (6) the Multi-Generation Register documents parental information on residents since 1961. Registers were linked using the personal identification number assigned to all residents at birth or migration to Sweden.

### Study Design

We conducted a nationwide, population-based matched cohort study. Using incidence density sampling, women of reproductive age with a first diagnosis of PMDs between January 1, 2001, and December 31, 2018, were randomly matched by year of birth to 5 women who were free of PMD at that date. Reproductive age was defined as the period between age 15 years (96% of Swedish women had menarche by then^17,18^) and 52 years (the average age of menopause in Sweden^17,18^). Women who had undergone hysterectomy or bilateral oophorectomy before matching were excluded from the analyses. Individuals were followed-up until death, emigration, or December 31, 2018, whichever came first. If matched unaffected women received a diagnosis of PMD during the study period, their follow-up was censored at that time (eFigure in Supplement 1).

We also conducted a sibling comparison to address unmeasured confounding from factors shared by sisters, such as early family environment and genetics. In brief, full siblings were identified as sharing both biological parents recorded in the Multi-Generation Register. Women with PMDs were matched to their full sisters on the basis of age at diagnosis such that the unaffected sister(s) had no PMD diagnosis at the same age when the affected sister received a diagnosis.

### Ascertainment of PMDs

As described elsewhere,^13^ PMDs were identified through inpatient and outpatient diagnoses, along with treatments. In brief, inpatient and outpatient diagnoses were identified using the Swedish version of *International Classification of Diseases, Ninth Revision (ICD-9) *or *International Statistical Classification of Diseases and Related Health Problems, Tenth Revision (ICD-10)*. The National Patient Register has nationwide coverage on inpatient care from 1987 onward and includes information on more than 80% of specialist-based outpatient visits from 2001 onward with high validity (positive predictive value of 85%-95% across diseases).^19^ To compensate for a lack of information on PMDs diagnosed in primary care, we identified prescriptions for antidepressant and oral contraceptives with a written indication for PMD treatment. Diagnoses and treatment codes are provided in eTable 1 in Supplement 1.

### Ascertainment of Deaths

Date and cause of death were obtained from the National Cause of Death Register. The underlying cause of death was classified according to *ICD-10 *codes as death due to nonnatural and natural causes (eTable 1 in Supplement 1). We further identified deaths due to neoplasms, cardiovascular diseases, and nervous system diseases as deaths from natural causes, whereas suicide and death due to accidents were classified as nonnatural causes. The register has a satisfactory accuracy rate for the studied causes of death (eg, 90% for malignant neoplasms, 87% for ischemic heart disease, and 96% for suicide).^20,21,22^ All deaths were considered as premature deaths because they occurred before the age of 70 years.^23^

### Covariates

Age at diagnosis or matching was calculated from date of birth, and country of birth was classified as born in Sweden or not. Region of residence, educational level, and income at the time of matching were derived from Longitudinal Integration Database for Health Insurance and Labor Market Studies, with regions grouped into south, center, or north of Sweden. Educational level was classified as 9 years or less, 10 to 12 years, more than 12 years, and unknown; personal income was divided into less than the 20th percentile, 20th to 80th percentile, and greater than the 80th percentile of the income distribution of the study population, and unknown.

Some psychiatric and somatic conditions are associated with both PMDs and premature death^8,10,11,12,24,25,26^ and were considered as confounders in this study. Psychiatric comorbidities were defined as any psychiatric diagnoses recorded for inpatient or outpatient specialist care visits by the time of matching. The Charlson Comorbidity Index, adapted to Swedish *ICD-9* or *ICD-10* codes,^27^ was used to assess somatic comorbidity. Only psychiatric and somatic comorbidities diagnosed before the matching date were included. Codes used to identify covariates are available in eTable 1 in Supplement 1.

### Statistical Analysis

Descriptive statistics were performed to summarize the baseline characteristics between women with and without PMDs. Next, hazard ratios (HRs) of all-cause mortality associated with PMDs and corresponding 95% CIs were estimated using Cox regression models conditional on the matching set for the population-matched cohort and the sibling set for the sibling-matched cohort. The underlying timescales were time since matching for the population-matched cohort and age at follow-up for the sibling-matched cohorts. The Schoenfeld residual-based test was used to confirm that there were no violations to the proportional-hazard assumption. HRs were adjusted for attained age (through matching or underlying timescale), educational level, residence, country of birth, and personal income and were additionally adjusted for somatic and psychiatric comorbidities. Stratification by age at diagnosis was conducted because early onset of PMDs may have a longer impact throughout reproductive ages. We also performed stratification by psychiatric and somatic comorbidities to examine potential risk modification.

Associations with mortality were further estimated for natural and nonnatural causes and for the major causes observed in the population. In corresponding analyses, individuals were censored for deaths due to causes other than the ones under study. Stratification by age at diagnosis was also conducted. Having seen comparable but underpowered results from sibling comparison in the main analysis, these analyses were performed in the population-matched cohort only.

Prospective symptom charting over 2 consecutive menstrual cycles is recommend when diagnosing PMDs.^28,29^ Because such information is not recorded in registers, we conducted a sensitivity analysis limited to women with a minimum of 2 recorded diagnoses of PMDs 28 days apart or longer. Owing to the challenges of determining intent in clinical practice, our main evaluation of suicide included deaths with undetermined intent,^30,31,32^ whereas in a sensitivity analysis we restricted to intentional self-harm (*ICD-10* codes X60-X84). Additional sensitivity analyses for deaths attributed to neoplasms or cardiovascular disease were restricted to individuals without a history of the corresponding disease. Because receiving a diagnosis of PMD can take several years,^33^ somatic and psychiatric comorbidities identified in the study could have occurred after PMDs onset and mediated the association with mortality. Allowing this possibility, we repeated the analyses without these comorbidities in the models. Finally, because treatment may influence natural mortality risk, we conducted stratifications by selective serotonin reuptake inhibitor (SSRI; Anatomical Therapeutic Chemical Classification N06AB) and hormonal replacement therapy (HRT; Anatomical Therapeutic Chemical Classification G03C and G03F) prescriptions, assessed as time-varying variables.

For all analyses, we used a 2-sided *P* < .05 to define statistical significance. Analyses were conducted from September 2022 to April 2023. Data were prepared in SAS statistical software version 9.4 (SAS Institute) and analyzed with Stata statistical software version 17.0 (StataCorp).

## Results

A total of 3 700 275 women were identified in the registers (eFigure in Supplement 1). Among them, 67 748 received a diagnosis of PMDs between 2001 and 2018 and were free from bilateral oophorectomy or hysterectomy at the time of PMD diagnosis. These women were matched at the time of diagnosis to 5 women fulfilling the same inclusion criteria and free from PMD (338 740 women), for a total population-matched cohort of 406 488 individuals. More than one-third of the women with PMDs had at least 1 full sister, and matching to up to 7 siblings resulted in a sibling-matched cohort of 55 801 individuals.

### Baseline Characteristics

The mean (SD) age at diagnosis or matching was 35.8 (8.2) years (Table 1). More than one-half of the women in the population-matched cohort resided in the middle of the country, reaching 65.4% (44 285 women) for women with PMDs. Somatic comorbidities were similarly distributed (6718 women with PMD [9.9%] and 30 870 women without PMD [9.1%] had ≥1 somatic comorbidity), whereas psychiatric disorders were more frequent among women with PMDs (16 160 women with PMD [23.8%] vs 47 919 women without PMD [14.1%]). Similar patterns were noted in the sibling-matched cohort.

**Table 1.** Characteristics of Population-Matched and Sibling-Matched Cohorts at Time of Matching

Characteristic	Women, No. (%)			
	Population-matched cohort		Sibling-matched cohort	
	With PMDs (n = 67 748)	Without PMDs (n = 338 740)	With PMDs (n = 24 789)	Without PMDs (n = 31 012)
Country of birth				
Sweden	61 757 (91.2)	304 073 (89.8)	23 956 (96.7)	29 406 (94.8)
Other	5991 (8.8)	34 667 (10.2)	833 (3.4)	1606 (5.2)
Age, y				
Mean (SD)	35.8 (8.2)	35.8 (8.2)	35.7 (8.2)	36.1 (8.3)
15-24	7074 (10.4)	35 375 (10.4)	2626 (10.6)	3150 (10.2)
25-34	20 960 (30.9)	104 939 (31.0)	7761 (31.3)	9498 (30.6)
35-44	29 131 (43.0)	145 520 (43.0)	10 577 (42.7)	12 803 (41.3)
45-51	10 583 (15.6)	52 906 (15.6)	3825 (15.4)	5561 (17.9)
Calendar year				
2001-2006	11 245 (16.6)	56 225 (16.6)	4874 (16.7)	9845 (31.7)
2007-2012	20 155 (29.7)	100 775 (29.7)	8332 (33.6)	11 008 (35.5)
2013-2018	36 348 (53.6)	181 740 (53.6)	11 583 (46.7)	10 159 (32.8)
Region of residence				
South	12 031 (17.8)	76 060 (22.4)	4624 (18.6)	6048 (19.5)
Middle	44 285 (65.4)	200 693 (59.2)	15 852 (63.9)	19 176 (61.8)
North	10 494 (15.5)	57 016 (16.8)	3985 (16.1)	5392 (17.1)
Unknown	938 (1.4)	4971 (1.5)	328 (1.3)	496 (1.6)
Educational level, y of education				
≤9	6422 (9.5)	34 096 (10.1)	2308 (10.4)	3236 (10.4)
10-12	28 227 (41.7)	145 824 (43.0)	10 685 (43.1)	13 811 (44.5)
>12	31 619 (46.7)	150 592 (44.5)	11 300 (45.6)	13 281 (42.8)
Unknown	1480 (2.2)	8228 (2.4)	496 (2.0)	684 (2.2)
Annual personal income, Swedish krona^a^				
<200 000	15 166 (22.4)	69 999 (20.7)	4259 (17.2)	5780 (18.6)
200 000 to 600 000	38 610 (57.0)	197 865 (58.4)	14 410 (58.1)	18 558 (59.84)
>600 000	12 889 (19.0)	65 010 (19.2)	5734 (23.1)	6124 (18.7)
Unknown	1083 (1.6)	5866 (1.7)	386 (1.6)	550 (1.8)
Presence of ≥1 comorbidities				
Somatic^b^	6718 (9.9)	30 870 (9.1)	2363 (9.5)	2579 (8.3)
Psychiatric	16 160 (23.8)	47 919 (14.1)	5375 (21.7)	4121 (13.3)

Abbreviation: PMD, premenstrual disorder.

^a^ As of April 17, 2024, $1 US = 10.94 Swedish krona.

^b^ Somatic comorbidities were assessed according to the Charlson Comorbidity Index score.

### All-Cause Mortality

During a mean (SD) follow-up of 6.2 (4.6) years (range, 1-18 years), 367 deaths were observed among women with PMDs (rate, 8.4 deaths per 10 000 person-years; 95% CI, 7.6-9.3 deaths per 10 000 person-years), and 1958 deaths were observed among women without PMDs (rate, 9.1 deaths per 10 000 person-years; 95% CI, 8.7-9.6 deaths per 10 000 person-years). Overall, women with PMDs did not have a higher risk of all-cause mortality compared with women without PMDs (age-adjusted HR, 0.91; 95% CI, 0.82-1.02) (Table 2). A lower mortality risk was found when accounting for demographics and comorbidities (HR, 0.88; 95% CI, 0.77-0.99). This lower risk was mainly seen among women with PMDs diagnosed at ages 45 to 51 years (HR, 0.79; 95% CI, 0.64-0.97), whereas women with PMDs diagnosed before the age of 25 years had more than doubled risk of death (HR, 2.51; 95% CI, 1.42-4.42). Largely comparable results were found in the sibling comparison (HR, 0.84; 95% CI, 0.67-1.12), although statistical power was limited, particularly in age-stratified analysis.

**Table 2.** All-Cause Mortality Among Women With PMDs Overall and by Age at Diagnosis or Matching

Variable	With PMDs		Without PMDs		Model 1^a^		Model 2^b^		Model 3^c^	
	Women, No.	IR^d^	Women, No.	IR^d^	HR (95% CI)	*P* value	HR (95% CI)	*P* value	HR (95% CI)	*P* value
Population-matched cohort										
Overall	367	8.4	1958	9.1	0.91 (0.82-1.02)	.26	0.92 (0.82-1.03)	.16	0.88 (0.77-0.99)	.04
By age at diagnosis or matching, y										
15-24	24	6.7	44	2.5	2.82 (1.70-4.68)	<.001	2.73 (1.61-4.61)	<.001	2.51 (1.42-4.42)	.001
25-34	68	5.9	261	4.6	1.28 (0.98-1.68)	.07	1.16 (0.87-1.55)	.30	1.01 (0.74-1.38)	.92
35-44	158	7.7	900	9.0	0.86 (0.72-1.02)	.08	0.87 (0.73-1.04)	.13	0.84 (0.70-1.01)	.06
45-51	117	14.9	753	19.3	0.76 (0.62-0-93)	.007	0.79 (0.65-0.97)	.02	0.79 (0.64-0.97)	.03
*P* for interaction	NA	NA	NA	NA	NA	<.001	NA	<.001	NA	.002
Sibling-matched cohort										
Overall	132	7.5	296	10.8	0.80 (0.62-1.04)	.10	0.85 (0.64-1.10)	.19	0.84 (0.67-1.12)	.18
By age at diagnosis or matching, y										
15-24	9	6.0	<5	1.3	4.06 (0.85-19.42)	.08	13.27 (1.25-140.94)	.03	9.20 (0.79-106.43)	.08
25-34	20	4.2	29	3.9	1.00 (0.51-1.96)	>.99	0.96 (0.50-1.97)	.92	1.08 (0.49-2.38)	.84
35-44	63	7.5	127	10.7	0.87 (0.61-1.26)	.48	0.98 (0.67-1.43)	.91	0.92 (0.61-1.39)	.70
45-51	40	12.7	137	23.7	0.54 (0.35-0.84)	.006	0.52 (0.35-0.84)	.008	0.54 (0.32-0.89)	.01
*P* for interaction	NA	NA	NA	NA	NA	.09	NA	.01	NA	.07

Abbreviations: HR, hazard ratio; IR, incidence rate; NA, not applicable; PMD, premenstrual disorder.

^a^ Model 1 is the crude HR with adjustment for age and year of birth by conditioning on the matching set in the population-based cohort and through direct adjustment in the sibling-matched cohort.

^b^ Model 2 was additionally adjusted for educational level, region of residence, country of birth, and personal income.

^c^ Model 3 was additionally adjusted for somatic comorbidity and psychiatric comorbidity. Somatic comorbidities were assessed according to the Charlson Comorbidity Index score.

^d^ The crude IR is defined as the number of events per 10 000 person-years.

The association of PMDs with all-cause mortality was comparable between women with and without psychiatric comorbidity (*P *for interaction = .58) (Table 3). However, the inverse association was greater among women with somatic comorbidities (HR, 0.47; 95% CI, 0.35-0.62). Again, this finding appeared to be more prevalent among women whose PMD was diagnosed at ages 45 to 51 years (eTable 2 in Supplement 1), whereas among women who received a diagnosis before age 25 years, there was no significant interaction with either comorbidity.

**Table 3.** All-Cause Mortality Among Women With PMDs in the Population-Matched Cohort, Stratified by Comorbidities^a^

Variable	With PMDs		Without PMDs		HR (95% CI)	*P* value	*P* for interaction
	Women, No.	IR^b^	Women, No.	IR^b^			
Psychiatric comorbidity							
Without	235	6.73	1467	7.67	0.90 (0.78-1.04)	.16	.58
With	132	15.34	491	21.50	1.05 (0.70-1.59)	.82	
Somatic comorbidity							
Without	299	7.51	1368	5.93	0.99 (0.88-1.13)	.97	<.001
With	68	18.32	590	35.10	0.47 (0.35-0.62)	<.001	

Abbreviations: HR, hazard ratio; IR, incidence rate; PMD, premenstrual disorder.

^a^ The model was adjusted for age, educational level, region of residence, country of birth, personal income, somatic comorbidity, and psychiatric comorbidity. Somatic comorbidities were assessed according to the Charlson Comorbidity Index score.

^b^ The crude IR is defined as the number of events per 10 000 person-years.

### Cause-Specific Mortality

The 5 most common causes of death were neoplasms (150 deaths [40.9%]), suicide (100 deaths [27.2%]), cardiovascular diseases (29 deaths [7.9%]), accident (29 deaths [7.9%]), and nervous system disease (18 deaths [4.9%]) (eTable 3 in Supplement 1). The PMD-associated risk of death due to natural causes followed the same pattern observed for all-cause mortality, with lower risk overall (HR, 0.73; 95% CI, 0.62-0.84) but elevated risk among women who received a diagnosis before the age of 25 years (HR, 2.59; 95% CI, 1.21-5.54) (Table 4). The lower risk was largely associated with cardiovascular-specific mortality (HR, 0.53; 95% CI, 0.34-0.84). There were 257 deaths attributed to cardiovascular disease, with 133 (51.8%) occurring in women who were included in the cohort at age 45 years or older (eTable 4 in Supplement 1). In contrast, mortality due to nonnatural causes was higher among women with PMDs (HR, 1.59; 95% CI, 1.24-2.04) and was primarily explained by suicide (HR, 1.92; 95% CI, 1.42-2.60). Notably, an elevated risk of suicide was observed regardless of the age at diagnosis (*P* for interaction = .68) (Table 5), although it was more pronounced among women who received a diagnosis before age 25 years (HR, 3.84; 95% CI, 1.18-12.45). Among women younger than 25 years who died, approximately one-third of the deaths (19 deaths) were due to suicide (eTables 4 and 5 in Supplement 1).

**Table 4.** Cause-Specific Mortality Among Women With PMDs Overall^a^

Cause of death	With PMDs		Without PMDs		HR (95% CI)	*P* value
	No.	IR^b^	No.	IR^b^		
Unnatural cause	129	2.96	355	1.65	1.59 (1.25-2.04)	<.001
Suicide	100	2.30	227	1.06	1.92 (1.42-2.60)	<.001
Accident	29	0.67	128	0.60	1.04 (0.65-1.66)	.86
Natural cause	238	4.46	1603	7.49	0.73 (0.62-0.84)	<.001
Neoplasm	150	0.34	932	0.43	0.88 (0.74-0.99)	.045
Cardiovascular disease	29	0.67	228	1.06	0.53 (0.34-0.84)	.006
Nervous system disease	18	0.41	85	0.39	0.86 (0.47-1.66)	.65

Abbreviations: HR, hazard ratio; IR, incidence rate; PMD, premenstrual disorder.

^a^ Models were adjusted for age, educational level, region of residence, country of birth, personal income, somatic comorbidity, and psychiatric comorbidity. Somatic comorbidities were assessed according to the Charlson score.

^b^ The crude IR is defined as the number of events per 10 000 person-years.

**Table 5.** Mortality Due to Suicide and Natural Deaths for Women With PMDs Overall and by Age^a^

Variable	With PMDs		Without PMDs		HR (95% CI)	*P* value
	Women, No.	IR^b^	Women, No.	IR^b^		
Suicide						
Overall	100	2.30	227	1.06	1.92 (1.42-2.60)	<.001
By age at diagnosis or matching, y						
15-24	9	2.52	10	0.56	3.84 (1.18-12.45)	.03
25-34	33	2.84	68	1.20	1.71 (1.01-2.89)	.045
35-44	38	1.85	103	1.02	1.95 (1.22-3.08)	.005
45-52	20	2.54	46	1.18	1.82 (0.92-3.64)	.09
*P* for interaction	NA	NA	NA	NA	NA	.68
Natural cause						
Overall	238	4.46	1603	7.49	0.73 (0.62-0.84)	<.001
By age at diagnosis or matching, y						
15-24	13	3.64	27	1.52	2.59 (1.21-5.54)	.01
25-34	30	2.58	161	2.89	0.79 (0.52-1.21)	.29
35-44	107	5.22	748	7.44	0.69 (0.55-0-85)	.001
45-52	88	11.18	667	17.08	0.68 (0.54-0.86)	.002
*P* for interaction	NA	NA	NA	NA	NA	.04

Abbreviations: HR, hazard ratio; IR, incidence rate; NA, not applicable; PMD, premenstrual disorder.

^a^ Models were adjusted for educational level, region of residence, country of birth, personal income, somatic comorbidity, and psychiatric comorbidity. Somatic comorbidities were assessed according to the Charlson Comorbidity Index score.

^b^ The crude IR is defined as the number of events per 10 000 person-years.

### Sensitivity Analyses

Comparable results were observed when the analysis was limited to PMDs with 2 diagnoses 28 days or more apart (eTable 6 in Supplement 1). Similarly, applying a stricter definition of suicide resulted in comparable estimates (eTable 7 in Supplement 1). Excluding individuals with a history of cancer or cardiovascular disease did not change the HR for the corresponding cause-specific mortality. Removing psychiatric or somatic comorbidities from regression models did not noticeably change the estimates (eTable 8 in Supplement 1). In addition, the inverse association between PMDs and natural mortality risk remained among individuals who did not use HRT or SSRI before or during the follow-up (eTable 9 in Supplement 1). SSRIs were prescribed to more than 80% of patients with PMD (55 552 women); 43% of women who received a diagnosis of PMDs at age 45 years or older were prescribed HRT (4110 women) compared with 23% of unaffected women (10 829 women). Finally, PMD-free women had a higher prevalence of diabetes or breast cancer (eTables 10 and 11 in Supplement 1).

## Discussion

This nationwide, population-based, matched cohort study with follow-up for up to 18 years fills an important gap in the understanding of all-cause and cause-specific mortality among women with PMDs. The use of national registers enabled complete follow-up and comprehensive information on death and its causes. Although no increased risk of all-cause death was observed overall, an elevated risk was noted among women who received a diagnosis before age 25 years. Women with PMDs were also found to have an elevated risk for suicide, regardless of age at diagnosis.

Our study revealed a consistently elevated risk of suicide among women with PMDs across all age groups. This is in line with previous research^14,34^ showing that individuals with PMDs have a higher prevalence of suicidality. Our recent study^13^ showed that women with PMDs were at increased risk of suicide attempt. However, to our knowledge, the current study is the first report to illustrate the increased risk of completed suicide.

Our findings further revealed that young women with diagnosed PMDs had an increased risk of all-cause mortality. Although suicidal behavior is more common in young women, this finding is not completely explained by suicide, which accounted for one-third of the deaths (eTables 4 and 5 in Supplement 1). Indeed, we also observed an increased risk of deaths due to natural causes in this group. Future studies with larger sample size are needed to examine the cause-specific mortality for these women.

In contrast, women who received a diagnosis at age 45 years or older had a lower risk of mortality than women without PMDs. These women may present a late-onset PMD or an exacerbation of mild symptoms, with a shorter cumulative impact of PMDs as symptoms are expected to end after menopause. We cannot exclude a potential healthy survivor effect because women who died before receiving a diagnosis or being identified in the registers were not included. Also, we must consider the potential for misclassification, as perimenopausal symptoms and depression may mirror PMDs symptoms, resulting in potential diagnostic errors. Moreover, women with premature menopause are not at risk of PMDs and might be oversampled in the unaffected group. Given that early menopause is associated with elevated risk of cardiovascular events^35,36^ and premature mortality,^37^ this may have influenced and partly explains the inverse associations. Specific information on menopause is lacking from registers; the age of first HRT prescription or diagnosis of early menopause were similar between the studied groups, although diagnoses and treatment rates are low in Sweden.^18^

The unexpected finding of a lower risk of death due to natural causes associated with PMDs, particularly from cardiovascular events, warrants further investigation. This association may have been influenced by the reduced mortality in women who received a diagnosis at 45 years or older, because 51.8% of the cardiovascular-specific deaths occurred among this group (eTable 4 in Supplement 1). Because PMDs are often underdiagnosed, women identified with PMDs may have a higher level of self-awareness and maintain closer contact with the health care system. They may be more inclined to behavior changes, receiving a diagnosis early, and/or receiving treatment for comorbidities. The lower risk of cardiovascular-specific death could also be related to the use of SSRIs, which were prescribed to more than 80% of the patients with PMD. The cardioprotective effect of SSRI has been reported, although they can confer adverse impact on specific cardiovascular diseases.^38,39^ A similar hypothesis can be formulated for HRT^40^: 43% of women who received a diagnosis of PMD at age 45 years or older were prescribed HRT compared with 23% for unaffected women. However, the inverse association remained among individuals not exposed to HRT or SSRIs. We also acknowledge the possibility of unexplored biases that could have influenced these unexpected findings, despite our concerted efforts to address them thoroughly.

PMDs are highly comorbid with psychiatric disorders,^8,9,12,41^ which are associated with a higher mortality risk.^12,40^ Although psychiatric comorbidities could have explained our findings among young women, we observed comparable associations in the presence or absence of psychiatric comorbidity. Notably, some women may develop major depression later in life,^9^ and such a mediation pathway warrants future research. Regarding somatic comorbidity, we found greater inverse associations between PMDs and mortality in the presence of 1 or more condition. This could be partially attributed a closer contact of affected women with the health care system. Moreover, although the prevalence for 1 or more comorbidity diagnosed before the matching date was comparable between women, the conditions and their severity may differ. For instance, some somatic comorbidities (eg, terminal stage of cancer) or treatments (eg, chemotherapy for breast cancer) can lead to amenorrhea or oligomenorrhea,^7,42,43,44^ reducing the occurrence of PMD symptoms. Indeed, in our data, PMD-free women had a higher prevalence of diabetes or breast cancer (eTables 10 and 11 in Supplement 1). The limited information on disease severity did not allow further investigation. The main strength of this study is the use of the Swedish register data, which allowed us to conduct a nationwide study with complete follow-up and comprehensive information on death and its causes.

### Limitations

This study has several limitations. First, although guidelines suggest diagnosis confirmation by a prospective evaluation of symptoms for 2 menstrual cycles,^29^ the registers lacked such information. However, restricting PMDs to 2 diagnoses registered separately yielded comparable results. Moreover, delayed diagnosis is frequent for PMDs,^45^ and most are diagnosed in primary care and not treated with medication, leading to potential misclassification of affected women as unaffected. However, such potential misclassification should not be related to the risk of death but may have attenuated the association toward the null.

Second, this study relied on recordings in the National Cause of Death Register, resulting in potential misclassification on the cause of death.^21^ A study^20^ showed an overall satisfactory accuracy rate of 77%, with variation depending on the disease (90% for malignant neoplasms, 87% for ischemic heart disease) and age (98% and 91% agreement in groups 0-44 and 45-64 years, respectively). We used *ICD-9* or *ICD-10* chapters to identify and group causes of death, minimizing the potential misclassification.^46^

Third, some potential confounding factors were not available in the registers (eg, smoking status^47,48^ or body mass index^25,49^). Comparable results were observed in the sibling comparison, which allowed us to address factors shared between sisters including familial environment, genetics, and possibly some lifestyle factors.

Fourth, the population under study was relatively young, with a mean (SD) age of 35.8 (8.2) years, and the mean (SD) duration of follow-up was 6.2 (4.6) years. As a result, the study primarily captured deaths due to nonnatural causes, and only short-term mortality could be assessed. The small number of events also limits the generalization of the results. Future studies with longer follow-up are needed to capture long-term consequences.

## Conclusion

Our findings suggest that women with clinically diagnosed PMDs were not at elevated risk of all-cause short-term death overall. However, women who received a diagnosis of PMD at an early age showed excess mortality, and the risk of suicide was elevated regardless of age. This supports the importance of careful follow-up for young women with PMDs and highlights the need to develop suicide prevention strategies for all women with PMDs.

## Notes

Supplement 1. **eFigure. **Flowchart: nationwide population-matched cohort and sibling-matched cohort, 2001-2018 **eTable 1.** Codes for identification of premenstrual disorders, psychiatric comorbidities, causes of death **eTable 2.** Hazard ratio (HR) and 95% confidence intervals (CI) of all-cause mortality among women with premenstrual disorders, stratified by age and comorbidities: population-based cohort 2001-2018 **eTable 3.** Incidence rates of death and distribution of underlying causes of death: population-matched cohort 2001-2018 **eTable 4.** Underlying cause of death by age at diagnosis: population-matched cohort 2001-2018, n=2,325 **eTable 5.** Distribution of the underlying causes of death for women younger than 25 years old: population-matched cohort 2001-2018, n=196 **eTable 6.** Sensitivity analysis: hazard ratio (HR) and 95% confidence interval (CI) of mortality for women with premenstrual disorders, overall and by age: nationwide population-matched cohort 2001-2018, PMDs defined with at least 2 diagnoses ≥28 days, N=192,618 **eTable 7.** Sensitivity analyses: hazard ratio (HR) and 95% confidence interval (CI) of cause-specific mortality: population-matched cohort 2001-2018 **eTable 8.** Hazard ratio (HR) and 95% confidence intervals (CI) of mortality among women with premenstrual disorders overall, by age at diagnosis/matching and by specific causes: nationwide population-matched cohort, 2001-2018 **eTable 9.** Hazard ratio (HR) and 95% confidence intervals (CI) of mortality among women with premenstrual disorders by hormone replacement therapy and selective serotonin inhibitor: nationwide population-matched cohort, 2005-2018 **eTable 10.** The most frequent comorbidities at matching, among individuals with at least one comorbidity, population-matched cohort 2001-2018 **eTable 11.** Distribution of the underlying causes of death for women diagnosed at age 45 or over, population-matched cohort 2001-2018

Supplement 2.
Data Sharing Statement
