# Error in Affiliations

**DOI:** 10.1001/jamanetworkopen.2024.24253

**Published:** 2024-06-20

In the Original Investigation titled “Mortality Risk Among Women With Premenstrual Disorders in Sweden,”^1^ published May 28, 2024, Dr Oberg’s affiliation was wrong. This article has been corrected.^1^

## References

[1] Opatowski M, Valdimarsdóttir UA, Oberg AS, Bertone-Johnson ER, Lu D. Mortality risk among women with premenstrual disorders in Sweden. JAMA Netw Open. 2024;7(5):e2413394. doi:10.1001/jamanetworkopen.2024.13394 38805225 PMC11134214

