# Supplementary material for: Mortality Risk Among Women With Premenstrual Disorders in Sweden
Source: JAMA Netw Open. 2024 May 28;7(5):e2413394. doi: 10.1001/jamanetworkopen.2024.13394 (PMC11134214; doi:10.1001/jamanetworkopen.2024.13394)
Supplement: Supplement 1. — eFigure. Flowchart: nationwide population-matched cohort and sibling-matched cohort, 2001-2018 eTable 1. Codes for identification of premenstrual disorders, psychiatric comorbidities, causes of death eTable 2. Hazard ratio (HR) and 95% confidence intervals (CI) of all-cause mortality among women with premenstrual disorders, stratified by age and comorbidities: population-based cohort 2001-2018 eTable 3. Incidence rates of death and distribution of underlying causes of death: population-matched cohort 2001-2018 eTable 4. Underlying cause of death by age at diagnosis: population-matched cohort 2001-2018, n=2,325 eTable 5. Distribution of the underlying causes of death for women younger than 25 years old: population-matched cohort 2001-2018, n=196 eTable 6. Sensitivity analysis: hazard ratio (HR) and 95% confidence interval (CI) of mortality for women with premenstrual disorders, overall and by age: nationwide population-matched cohort 2001-2018, PMDs defined with at least 2 diagnoses ≥28 days, N=192,618 eTable 7. Sensitivity analyses: hazard ratio (HR) and 95% confidence interval (CI) of cause-specific mortality: population-matched cohort 2001-2018 eTable 8. Hazard ratio (HR) and 95% confidence intervals (CI) of mortality among women with premenstrual disorders overall, by age at diagnosis/matching and by specific causes: nationwide population-matched cohort, 2001-2018 eTable 9. Hazard ratio (HR) and 95% confidence intervals (CI) of mortality among women with premenstrual disorders by hormone replacement therapy and selective serotonin inhibitor: nationwide population-matched cohort, 2005-2018 eTable 10. The most frequent comorbidities at matching, among individuals with at least one comorbidity, population-matched cohort 2001-2018 eTable 11. Distribution of the underlying causes of death for women diagnosed at age 45 or over, population-matched cohort 2001-2018 [file jamanetwopen-e2413394-s001.pdf]

Supplemental Online Content

Opatowski M, Valdimarsdóttir UA, Oberg AS, Bertone-Johnson ER, Lu D. Mortality risk among women with premenstrual disorders in Sweden. *JAMA Netw Open*. 2024;7(5):e2413394. doi:10.1001/jamanetworkopen.2024.13394

eFigure. Flowchart: nationwide population-matched cohort and sibling-matched cohort, 2001-2018

eTable 1. Codes for identification of premenstrual disorders, psychiatric comorbidities, causes of death

eTable 2. Hazard ratio (HR) and 95% confidence intervals (CI) of all-cause mortality among women with premenstrual disorders, stratified by age and comorbidities: population-based cohort 2001-2018

eTable 3. Incidence rates of death and distribution of underlying causes of death: population-matched cohort 2001-2018

eTable 4. Underlying cause of death by age at diagnosis: population-matched cohort 2001-2018, n=2,325

eTable 5. Distribution of the underlying causes of death for women younger than 25 years old: population-matched cohort 2001-2018, n=196

eTable 6. Sensitivity analysis: hazard ratio (HR) and 95% confidence interval (CI) of mortality for women with premenstrual disorders, overall and by age: nationwide population-matched cohort 2001-2018, PMDs defined with at least 2 diagnoses ≥28 days, N=192,618

eTable 7. Sensitivity analyses: hazard ratio (HR) and 95% confidence interval (CI) of cause-specific mortality: population-matched cohort 2001-2018

eTable 8. Hazard ratio (HR) and 95% confidence intervals (CI) of mortality among women with premenstrual disorders overall, by age at diagnosis/matching and by specific causes: nationwide population-matched cohort, 2001-2018

eTable 9. Hazard ratio (HR) and 95% confidence intervals (CI) of mortality among women with premenstrual disorders by hormone replacement therapy and selective serotonin inhibitor: nationwide population-matched cohort, 2005-2018

eTable 10. The most frequent comorbidities at matching, among individuals with at least one comorbidity, population-matched cohort 2001-2018

eTable 11. Distribution of the underlying causes of death for women diagnosed at age 45 or over, population-matched cohort 2001-2018

This supplemental material has been provided by the authors to give readers additional information about their work.

© 2024 Opatowski M et al. *JAMA Network Open.*

eFigure. Flowchart: nationwide population-matched cohort and sibling-matched cohort, 2001-2018

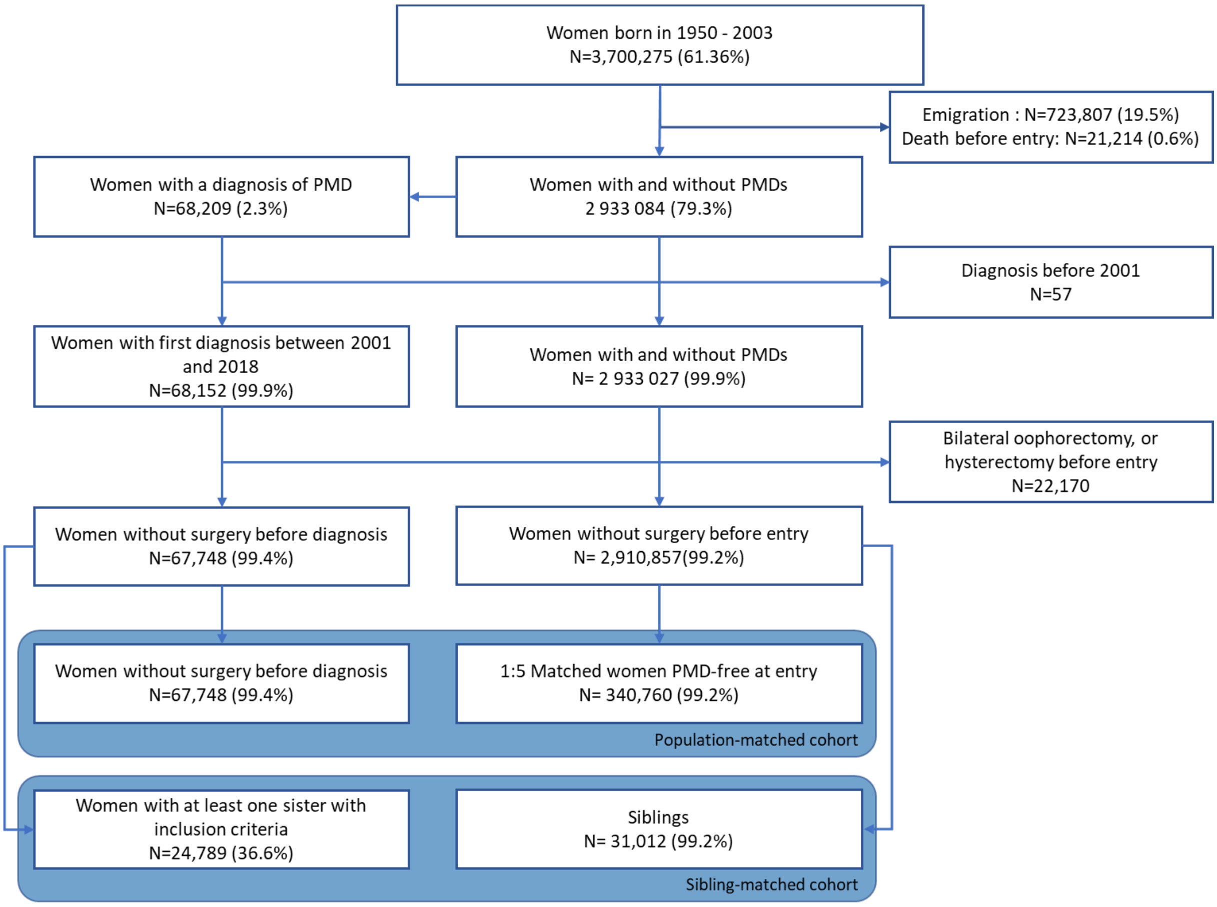

Abbreviations: PMDs, premenstrual disorders

© 2024 Opatowski M et al. *JAMA Network Open.*

eTable 1. Codes for identification of premenstrual disorders, psychiatric comorbidities, causes of death

Diagnosis	Source	Type	Codes
Premenstrual disorders	NPR	ICD-10	N943 ,625E
	MDR	ATC	N06AB, N06AX, N06AA, G03A, G02B *Associated with a Swedish written indication for PMDs: "PMS" , "PREMENSTRUELLT SYNDROM", "PREMENSTRUELLT DYSFORSIKT SYNDROM", "PREMENSTRUELLT DYSFORI", "PMD", "PMDD", "PMDS", "MENS"*
Psychiatric comorbidity	NPR	ICD-9	291, 292, 295-311
		ICD-10	F10- F69
Oophorectomy and hysterectomy	NPR	ICD-9 procedure codes	7021, 7031, 7210, 7211, 7261, 7262, 7467
		ICD-10 procedure codes	LAE20, LAF10, LCD00, LCC10, LCD10, LCD11, LCD01, LCD04, LEF13
Charlson Index	NPR	ICD-8, 9 and 10	*Adaptation of the Charlson Comorbidity Index for Register-Based Research in Sweden*[1]
Residency	LISA	County	
South			6, 7, 8, 9, 10, 11, 12
Middle			1, 2, 3, 4, 5, 13, 14, 15, 16, 18, 19
North			17, 20, 21, 22, 23, 24, 25
Death Overall	NDR	ICD-10	All chapters
Neoplasms			C00-C99
Cardiovascular system			I00-I99
Suicide			X60-X84, Y10-Y34, Y870
Accident			V01-X59, Y85-Y86 (except Y870)
Nervous system			G00-G99
Unnatural death			S00-Y98

Abbreviations: ICD, International Classification of Diseases; LISA, Longitudinal Integration Database for Health Insurance and Labor Market Studies; MDR, National Medical Drug Register; NDR, National Cause of Death Register; PMDs, Premenstrual Disorders

[1] ICD codes codes provided by *Ludvigsson JF, Appelros P, Askling J, et al. Adaptation of the charlson comorbidity index for register-based research in sweden. Clin Epidemiol. 2021;13:21-41*

eTable 2. Hazard ratio (HR) and 95% confidence intervals (CI) of all-cause mortality among women with premenstrual disorders, stratified by age and comorbidities: population-based cohort 2001-2018

	With PMDs n (IR)	Without PMDs n (IR)	HR [95% CI]	p	Interaction
Age at diagnosis/matching, < 25					
By psychiatric comorbidity					p=0.498
Without	12 (14.33)	14 (7.44)	2.18 [1.04 – 4.57]	0.038	
With	12 (4.38)	30 (1.90)	3.78 [1.00 – 14.26]	0.050	
By somatic comorbidity					p=0.695
Without	20 (6.25)	33 (2.01)	2.66 [1.39 – 5.09]	0.003	
With	4 (10.69)	11 (8.40)	1.79 [0.29 – 10.98]	0.527	
Age at diagnosis/matching, 45 - 52					
By psychiatric comorbidity					p=0.929
Without	80 (12.13)	580 (15.14)	0.79 [0.62 – 1.02]	0.068	
With	37 (28.87)	173 (46.21)	0.78 [0.50 – 1.22]	0.268	
By somatic comorbidity					p=0.017
Without	98 (13.76)	512 (14.52)	0.89 [0.71 –1.11]	0.324	
With	19 (25.18)	241 (63.43)	0.42 [0.23 – 0.75]	0.003	

Abbreviations: CI, Confidence Interval; HR: Hazard Ratio; IR, Crude incidence rate, number of cases per 10,000 person-years; n, number of events; p, p-value; PMDs, Premenstrual disorders

The models were adjusted for age, educational level, region of residence, country of birth, personal income, somatic comorbidity, and psychiatric comorbidity. Somatic comorbidities were assessed based on the Charlson score.

© 2024 Opatowski M et al. *JAMA Network Open.*

eTable 3. Incidence rates of death and distribution of underlying causes of death: population-matched cohort 2001-2018

	With PMDs N=67,748	Without PMDs N=338,740
Death during follow-up		
By PMD (n,%)	367 (0.54)	1,958 (0.58)
Incidence rate [95% CI] / 10,000 PYs	8.43 [7.61– 9.34]	9.14 [8.75–9.56]
Causes of death, distribution n(%)		
Neoplasm	150 (40.87)	951 (48.57)
Cardiovascular disease	29 (7.90)	228 (11.64)
Suicide	100 (27.25)	227 (11.59)
Accident	29 (7.90)	128 (6.54)
Nervous system disease	18 (4.90)	85 (4.34)
Digestive system disease	8 (2.18)	84 (4.29)
Abnormal tests	8 (2.18)	59 (3.01)
Endocrine system disease	5 (1.36)	44 (2.25)
Respiratory system disease	4 (1.09)	42 (2.15)
Mental and behaviors disorder	2 (0.54)	26 (1.33)
Congenital disease	1 (0.27)	26 (1.33)
Infection	6 (1.63)	21 (1.07)
Others	7 (1.91)	37 (1.89)
Three most frequent cancers causing death, distribution n(%)		
Malignant neoplasm of breast	237 (25.43)	37 (24.67)
Malignant neoplasm of bronchus and lung	114 (12.23)	18 (12.00)
Malignant neoplasm of brain	61 (7.51)	9 (6.00)

Abbreviations: n, number of events; PMDs, Premenstrual disorders; PYs, Person-years

© 2024 Opatowski M et al. *JAMA Network Open.*

eTable 4. Underlying cause of death by age at diagnosis: population-matched cohort 2001-2018, n=2,325

Cause of death	Age at diagnosis<br>15-24<br>n (%)	 25 – 34<br>n (%)	 35 – 44<br>n (%)	 45 – 52<br>n (%)
Suicide	19 (27.94)	101 (30.70)	141 (13.33)	66 (7.59)
Cardiovascular diseases	5 (7.35)	24 (7.29)	95 (8.98)	133 (15.29)
Other	44 (64.71)	204 (62.01)	822 (77.69)	671 (77.13)

eTable 5. Distribution of the underlying causes of death for women younger than 25 years old: population-matched cohort 2001-2018, n=196

Cause of death	With PMDs		Without PMDs	
	n (%)	IR	n (%)	IR
Neoplasms	3 (12.50)	0.84	141 (25.00)	7.97
Suicide	9 (37.50)	2.52	10 (22.73)	0.56
Accident	2 (8.33)	0.56	7 (15.91)	0.39
Nervous system disease	5 (20.83)	1.40	2 (4.55)	0.11
Cardiovascular disease	1 (4.17)	0.28	4 (9.09)	0.22
Abnormal tests	1 (4.17)	0.28	3 (6.82)	0.17
Congenital disease	1 (4.17)	0.28	2 (4.55)	0.11
Endocrine system disease	1 (4.17)	0.28	1 (2.27)	0.06
Infection	0	-	2 (4.45)	0.11
Digestive system disease	0	-	1 (2.27)	0.06
Musculoskeletal system disease	0	-	1 (2.27)	0.06
Respiratory system disease	1 (4.17)	0.28	0	-

Abbreviations: IR, Crude incidence rate, number of cases per 10,000 person-years; n, number of events; PMDs, Premenstrual disorders

eTable 6. Sensitivity analysis: hazard ratio (HR) and 95% confidence interval (CI) of mortality for women with premenstrual disorders, overall and by age: nationwide population-matched cohort 2001-2018, PMDs defined with at least 2 diagnoses ≥28 days, N=192,618

Death	With PMDs n (IR)	Without PMDs n (IR)	HR	95% CI	p
**Overall**	120 (6.23)	735 (7.77)	0.80	[0.65 – 0.99]	0.042
**By age at diagnosis or entry**					
15-24 years old	7 (4.34)	15 (1.88)	2.57	[0.92 – 7.20]	0.072
25-34 years old	32 (5.48)	125 (4.38)	1.21	[0.77 – 1.92]	0.399
35-44 years old	54 (5.98)	362 (8.16)	0.71	[0.52 – 0.97]	0.034
45-52 years old	27 (9.73)	233 (16.93)	0.66	[0.43 – 1.01]	0.060
**Suicide**	37 (1.92)	92 (0.97)	2.19	[1.30 – 3.67]	0.003

Abbreviations: CI, Confidence Interval; HR, Hazard Ratio; IR, Crude incidence rate, number of cases per 10,000 person-years; n, number of events; p, p-value; PMDs, Premenstrual disorders

The models were adjusted for educational level, region of residence, country of birth, personal income, somatic and psychiatric comorbidities. Somatic comorbidities were assessed based on the Charlson score

eTable 7. Sensitivity analyses: hazard ratio (HR) and 95% confidence interval (CI) of cause-specific mortality: population-matched cohort 2001-2018

Causes for death	With PMDs n (IR)	Without PMDs n (IR)	HR	95% CI	p
**Suicide restricted to diagnoses of self-harm**[1]					
Self-harm	82 (1.88)	171 (0.80)	2.10	[1.51 – 2.91]	<.001
**Exclusion of women with history of cancer**[2]					
Neoplasms	932 (0.43)	150 (0.34)	0.93	[0.77 – 1.12]	0.448
**Exclusion of women with history of cardiovascular diseases**[3]					
Cardiovascular diseases	177 (0.85)	24 (0.56)	0.53	[0.33 – 0.86]	0.015

Abbreviations: CI, Confidence Interval; HR, Hazard Ratio; IR, Crude incidence rate, number of cases per 10,000 person-years; n, number of events; p, p-value; PMDs, Premenstrual disorders

The models were adjusted for age, educational level, region of residence, country of birth, personal income, somatic comorbidity, and psychiatric comorbidity. Somatic comorbidities were assessed based on the Charlson score

[1] The definition of suicide excluded undetermined intent. N total population=406,488

[2] The women with an history of cancer before PMD diagnosis/matching were excluded from the analysis. N total population=399,881

[3] The women with an history of cardiovascular disease before PMD diagnosis/matching were excluded from the analysis, N total population=394,085

© 2024 Opatowski M et al. *JAMA Network Open.*

eTable 8. Hazard ratio (HR) and 95% confidence intervals (CI) of mortality among women with premenstrual disorders overall, by age at diagnosis/matching and by specific causes: nationwide population-matched cohort, 2001-2018

	With PMDs n (IR)	Without PMDs n (IR)	Model 4 HR [95% CI]	p	Model 5 HR [95% CI]	p
All cause	367 (8.4)	1958 (9.1)	0.95 [0.84 – 1.07]	0.397	0.84 [0.75 – 0.95]	0.004
By age at diagnosis/matching						
15-24	24 (6.7)	44 (2.5)	2.39 [1.39 – 4.13]	0.002	2.84 [1.64 – 4.91]	<.001
25-34	68 (5.9)	261 (4.6)	1.01 [0.76 – 1.35]	0.948	1.15 [0.86 – 1.54]	0.336
35-44	158 (7.7)	900 (9.0)	0.81 [0.68 – 0.97]	0.020	0.89 [0.74 – 1.06]	0.188
45-51	117 (14.9)	753 (19.3)	0.72 [0.59 – 0.88]	0.002	0.83 [0.68 – 1.03]	0.092
Interaction				<.001		<.001
Unnatural cause	129 (2.96)	355 (1.65)	1.59 [1.24 – 2.04]	<.001	1.92 [1.54 – 2.39]	<.001
Suicide	100 (2.30)	227 (1.06)	1.92 [1.44 – 2.60]	<.001	2.40 [1.84 – 3.10]	<.001
Accident	29 (0.67)	128 (0.60)	1.17 [0.77 – 1.80]	0.485	1.04 [0.65 – 1.65]	0.874
Natural cause	238 (4.46)	1603 (7.49)	0.69 [0.60 – 0.80]	<.001	0.75 [0.65 – 0.87]	<.001
Neoplasm	150 (0.34)	932 (0.43)	0.77 [0.65 – 0.92]	0.004	0.82 [0.69 – 0.99]	0.035
Cardiovascular disease	29 (0.67)	228 (1.06)	0.50 [0.32 – 0.78]	0.002	0.59 [0.38 – 0.90]	0.017
Nervous system disease	18 (0.41)	85 (0.39)	0.96 [0.54 – 1.70]	0.883	0.88 [0.48 – 1.60]	0.673

Abbreviations: IR, Crude incidence rate, number of events per 10,000 person-years; n, number of events; PMDs, Premenstrual disorders

Model 4, no adjustment for somatic comorbidities: Models adjusted for age and year of birth by conditioning on the matching set in the population-based cohort and through direct adjustment in the sibling-matched cohort, educational level, region of residence, country of birth, personal income and psychiatric comorbidity.

Model 5, no adjustment for psychiatric comorbidities: Models adjusted for age and year of birth by conditioning on the matching set in the population-based cohort and through direct adjustment in the sibling-matched cohort, educational level, region of residence, country of birth, personal income and somatic comorbidity. Somatic comorbidities were assessed based on the Charlson score.

eTable 9. Hazard ratio (HR) and 95% confidence intervals (CI) of mortality among women with premenstrual disorders by hormone replacement therapy and selective serotonin inhibitor: nationwide population-matched cohort, 2005-2018

	With PMDs n (IR)	Without PMDs n (IR)	HR [95% CI]	p	Interaction
Natural deaths					
By HRT before and during follow-up					
Without	118 (3.67)	973 (5.84)	0.68 [0.55 – 0.84]	<0.001	0.320
With	43 (13.87)	133 (18.18)	0.86 [0.58 – 1.28]	0.465	
By SSRI before and during follow-up					
Without	129 (4.17)	932 (5.76)	0.75 [0.62 – 0.92]	0.005	0.393
With	32 (7.33)	174 (14.46)	0.60 [0.38 – 0.95]	0.031	

Abbreviations: HRT, Hormone Replacement Therapy ; SSRI, Selective Serotonin Inhibitor

HRT and SSRI were considered as time-varying covariates

© 2024 Opatowski M et al. *JAMA Network Open.*

eTable 10. The most frequent comorbidities at matching, among individuals with at least one comorbidity, population-matched cohort 2001-2018

	With PMDs n (%)	Without PMDs n (%)
Asthma	3,724 (55.43)	15,165 (49.12)
Kidney disease	593 (8.83)	2349 (7.76)
Diabetes	578 (8.60)	3778 (12.23)
Systemic connective tissue disorders	574 (8.54)	2572 (8.33)
Malignant neoplasms of bone and articular cartilage	442 (6.58)	2056 (6.66)
Arthropathies	368 (5.48)	2196 (7.11)
Viral hepatitis	329 (4.90)	1568 (5.07)
Malignant neoplasm of breast	253 (3.77)	2046 (6.63)

Abbreviations: n, number of individuals with the comorbidity; PMDs, premenstrual disorders

Note: One individual could have several comorbidities, therefore the sum of percentages can exceed 100%.

eTable 11. Distribution of the underlying causes of death for women diagnosed at age 45 or over, population-matched cohort 2001-2018

Cause of death	With PMDs		Without PMDs	
	n (%)	IR	n (%)	IR
Neoplasms	58 (49.57)	7.36	393 (52.19)	10.06
Suicide	20 (17.09)	2.54	46 (6.11)	1.18
Accident	9 (7.69)	1.14	40 (5.31)	1.02
Nervous system disease	3 (2.56)	0.38	34 (4.52)	0.87
Cardiovascular disease	14 (11.97)	1.77	121 (16.07)	3.09
Abnormal tests	3 (2.56)	0.38	13 (1.73)	0.33
Congenital disease	0	-	7 (0.93)	0.18
Endocrine system disease	0	-	21 (2.79)	0.54
Infection	3 (2.56)	0.38	10 (1.33)	0.26
Digestive system disease	2 (1.71)	0.25	39 (5.18)	0.99
Musculoskeletal system disease	1 (0.85)	0.13	4 (0.53)	0.10
Respiratory system disease	2 (1.17)	0.25	17 (2.26)	0.43
Mental and behavior disorders	1 (0.85)	0.13	5 (0.66)	0.13
Other	1 (0.13)	0.13	2 (0.27)	0.51

Abbreviations: IR, Crude incidence rate, number of cases per 10,000 person-years; n, number of events; PMDs, Premenstrual disorders

© 2024 Opatowski M et al. *JAMA Network Open.*
