# Supplementary material for: Mortality Risk Among Women With Premenstrual Disorders in Sweden
Source: JAMA Netw Open. 2024 May 28;7(5):e2413394. doi: 10.1001/jamanetworkopen.2024.13394 (PMC11134214; doi:10.1001/jamanetworkopen.2024.13394)
Supplement: Supplement 2. — Data Sharing Statement [file jamanetwopen-e2413394-s002.pdf]

# Data Sharing Statement

Opatowski. Mortality Risk Among Women With Premenstrual Disorders in Sweden. *JAMA Netw Open*. Published May 28, 2024. doi:10.1001/jamanetworkopen.2024.13394

**Data**
**Data available:** No

**Additional Information**
**Explanation for why data not available:** Register individual patient data cannot be shared
